# Helicobacter Pylory infection in patients with esophageal squamous cell carcinoma

**DOI:** 10.6061/clinics/2017(03)04

**Published:** 2017-03

**Authors:** Omer Bilgehan Poyrazoglu, Ahmet Cumhur Dulger, Bilge Sumbul Gultepe

**Affiliations:** I Lokman Hekim Hospital, General Surgery, Van, Turkey; II Yuzuncu Yil University, School of Medicine, Department of Gastroenterology, Van, Turkey; III Bezmialem Vakif University, School of Medicine, Microbiology, Istanbul, Turkey

**Keywords:** Helicobacter pylori, Esophageal Squamous Cell Carcinoma, Turkey

## Abstract

**OBJECTIVE:**

Esophageal squamous cell carcinoma is one of the most common esophageal diseases in the developing world, but the relationship between esophageal squamous cell carcinoma and Helicobacter pylori infection remains a neglected topic. The primary objective of this study was to determine the association between Helicobacter pylori infection and esophageal squamous cell carcinoma. A second purpose was to determine the incidence and factors associated with Helicobacter pylori infection following esophagectomy.

**METHOD:**

The microorganism was identified by testing the gastric biopsy materials from 95 esophageal squamous cell carcinoma patients (66 females; 39 were esophagectomized) for urease activity in a medium containing urea and a power of hydrogen detection reagent and comparing the results with those from a healthy population. Differences in patient characteristics were assessed with chi-square tests and t-tests for categorical and continuous factors, respectively.

**RESULTS:**

The patients with esophageal squamous cell carcinoma had a significantly lower prevalence of Helicobacter pylori compared with the healthy population (*p*<0.001). The naive and esophagectomized patients, in contrast, showed no significant differences in Helicobacter pylori infection (*p*>0.005). Patients with esophageal squamous cell carcinoma showed a significant association between leukocytosis and hypoglobulinemia and the presence of Helicobacter pylori infection (*p*=0.023 and *p*=0.045, respectively).

**CONCLUSION:**

These results suggest that Helicobacter pylori is not an etiological factor in patients with esophageal squamous cell carcinoma. We found a statistically significant negative correlation between esophageal squamous cell cancer and Helicobacter pylori infection. These findings may guide new strategies for esophageal squamous cell carcinoma therapy.

## INTRODUCTION

Esophageal squamous cell carcinoma (ESCC), one of the most aggressive digestive system tumors, is associated with numerous factors, including advanced age, achalasia, Plummer-Vinson syndrome, low socioeconomic status, high-starch diets lacking in fruits and vegetables, alcohol abuse, tobacco use, previous head and neck squamous cell carcinoma, and radiation therapy 1. It is also an important cause of mortality in the Asian esophageal cancer belt and in the eastern part of Turkey 2.

Helicobacter pylori (HP), a gram-negative bacterium found on the gastric mucosa, was first isolated 30 years ago 3. Infection with HP is one cause of duodenal or gastric ulcers (reported to develop in 1 to 10% of infected patients), gastric cancer (in 0.1 to 3%), and gastric mucosa-associated lymphoid-tissue (MALT) lymphoma (in <0.01%) 4. Recent studies have demonstrated a high frequency of HP infection in gastric cancer patients, suggesting that presence of HP may function as a driver of the events contributing to oncogenesis in gastric adenocarcinomas 5,6. Furthermore, an inverse association has been established between Cag A-positive HP infection and the risk of esophageal adenocarcinoma 7. HP infection plays crucial roles in gastric carcinogenesis; however, the impact of HP on ESCC is not well understood.

The available data, derived from studies using serologic tests, are conflicting with respect to any association between HP and ESCC 8-10. The aim of the present study was to examine the potential correlation between ESCC and HP infection and to compare the presence of HP in naive ESCC patients and their esophagectomized counterparts.

## PATIENTS AND METHODS

This retrospective trial was conducted at a university medical center in a large metropolitan area near the Iranian border of Turkey, where both ESCC and HP infection are endemic. In total, 95 ESCC patients (65 women, aged 32-92 years) were evaluated in our clinic from July 2012 to July 2015. All the patients were diagnosed with ESCC based on established endoscopic and histopathological criteria. Of these, 39 had undergone a subtotal esophagectomy. In this esophagectomy group, esophageal reconstruction was performed through a subcutaneous route in 10 patients, through a retrosternal route in 25, and through a posterior mediastinal route in 4. The reconstructed esophagus was a wide gastric tube, as described by Holscher 11.

Postoperatively, the patients typically underwent upper gastrointestinal endoscopy 6 weeks after surgery to determine the presence or absence of HP infection.

Gastric biopsy samples were tested for urease activity using a commercial Hp-fast test kit (GI Supply®, Camp Hill, PA, USA) consisting of a urea-containing medium and a power of hydrogen (pH) detection reagent.

The control group comprised 151 dyspeptic subjects (100 women and 51 men, aged 30–85 years). Controls were also required to be medical-treatment free for at least 6 months from the time of study entry. Antrum biopsies with normal endoscopic evaluations were examined for HP using the same method. The prognostic values of the presence of HP and other clinicopathologic factors were also evaluated.

### Statistical analysis

The two groups were compared using the Mann-Whitney U-test. The differences were considered statistically significant at *p*<0.005.

## RESULTS

The mean age was 52.96±11.81 years in the control group (100 women), 59.53±13.93 years in the naive ESCC group (36 females), and 55.95±11.53 years in esophagectomized group (28 females). Nearly two-thirds of the ESCC patients were female. Descriptive statistics and comparison results according to the presence of HP are shown in Table 1.

HP infection was observed in 39 (68.4%) of the 57 naive ESCC patients, 27 (69.2%) of the 39 esophagectomized patients, and 128 (85%) of the 151 dyspeptic control patients (Figure 2). We found a significantly lower rate of HP in patients with ESCC compared with the dyspeptic subjects (*p*<0.0001), whereas no statistically significant difference was detected between the naive ESCC patients and their esophagectomized counterparts (*p*>0.005). We also found no gender differences between the groups (*p*>0.0005).

Significantly higher levels of leukocytes and serum globulin were also found in the ESCC patients diagnosed with HP compared with those without HP infection (*p*=0.023 for leukocytes and *p*=0.045 for serum globulin).

## DISCUSSION

Our study revealed a strong association between HP infection and a reduced risk of ESCC. In the Turkish adult population, the prevalence of HP infection is higher than reported in Western countries. A previous investigation of 4622 Turkish subjects indicated an HP infection prevalence of 82.5% 12. The prevalence of HP infection in the present study was similar (85%). Squamous cell carcinomas are usually detected in the proximal two-thirds of the esophagus. They predominantly affect elderly people and usually present as dysphagia, odynophagia, and unintentional weight loss 13. Worldwide, esophageal cancer ranks fifth in mortality among all malignancies, and ESCC remains the most common type.

In Asia, upper gastrointestinal cancers constitute a major group of malignancies with high rates of morbidity and mortality. The esophageal cancer belt, originating in the Far East and extending through middle Asia and the Near East, encompasses many countries, including northern China, northern Iran and the eastern part of Turkey 14. The predominant histopathological type of esophageal cancer is the squamous cell type in the endemic Asian regions, with incidence rates that may vary 200-fold among different populations within the same defined region due to cultural practices. More than 80% of ESCC patients in the rural areas of Asia also present at advanced stages that are not amenable to curative therapies; hence the need for novel preventive strategies is urgent 15.

The Van region of eastern Turkey is located in the western end of the esophageal cancer belt. Esophageal and gastric cancers are the most prevalent malignancies in both females and males in eastern part of Turkey 16. The probable culprit factors for ESCC in this region are low educational and socioeconomic status; the consumption of smoked, salted, hot, fatty foods; overconsumption of hot tea; cigarette smoking; poor intake of fresh fruits and vegetables, and poor hygienic conditions 17. Previous studies have shown that the eastern part of Turkey has one of the highest rates of both ESCC and HP infection. An HP infection rate of 36% was reported for gastric biopsy specimens in patients with gastric carcinoma from the Van region 18.

Various environmental factors, including cigarette smoking and excessive alcohol intake, can be associated with an increased risk of developing ESCC 19. HP is considered one of the most important human carcinogens for the upper gastrointestinal tract and the stomach 20. At present, a few reports have indicated a possible relationship between HP and ESCC, but most of these were performed using non-endoscopic (serologic) techniques 7. A recent Chinese study reported an HP seropositivity of 35.3% in ESCC patients, which was lower than that of the control groups (40% and 59%) 21. A recent meta-analysis also found an association between CagA-negative HP strains and a marginally significant increased risk of ESCC 7. In contrast, a prospective and serological study from China showed no association between HP and ESCC 22. Another recent meta-analysis from China showed an association between HP infection and a decreased risk of ESCC in Eastern populations and a decreased risk of esophageal adenocarcinoma (EAC) in the overall population 23.

**Figure 1:**
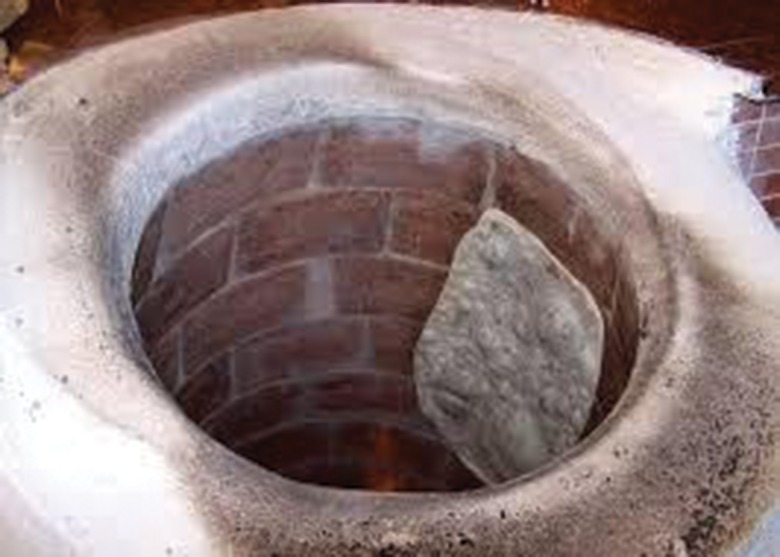
Traditional large oven (Tandir).

**Figure 2:**
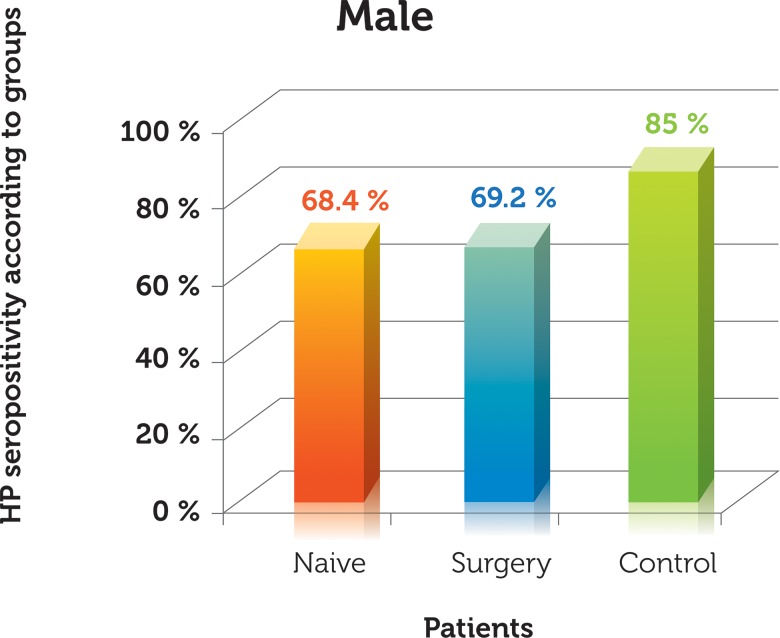
Rate of HP according to groups.

The current study revealed no association between HP infection and ESCC among people living in the eastern part of Turkey. The low prevalence of HP in these ESCC patients was similar to that reported in the Chinese meta-analyses. We also found a predominance of ESCC in female patients, in agreement with a previous Turkish study 17. This phenomenon has been linked to the use of the traditional large ovens (tandirs) that use smoke to cook meals (Figure 1). The mechanisms by which these ovens induce esophageal carcinogenesis remain undefined, but previous work suggests that smoke may play role similar to that of cigarette smoking 17. We conclude that an effective follow-up strategy for HP-negative female Asian adults will be necessary if ESCC screening is to yield public health benefits. The higher levels of leucocytes and serum globulin among ESCC patients with HP may also reflect an emerging phenomenon that requires additional investigation to determine the underlying causative factors.

The H. pylori-fast test (Hp-fast test) is based on the detection of HP urease activity and has a high sensitivity (85%) and specificity (>95%) for detecting HP infection. As this test is considered cost-effective and suitable for endoscopy units 24, we used the Hp-fast test to establish HP infection.

The current data are limited regarding any changes in the prevalence of HP in patients who have undergone esophagectomy. We therefore performed gastroduodenoscopy with pathological examination of the biopsy specimens obtained from the gastric conduit. We observed that the rate of HP infection was lower in esophagectomized ESCC patients than in the control subjects (19% and 78%, respectively; *p*<0.001). The rate of HP infection was similar, however, between the naive ESCC patients and the esophagectomized patients (*p*>0.005). A Japanese study reported that the HP infection status changed from preoperatively positive to postoperatively negative and that this changing pattern was linked to the eradication of HP via the perioperative administration of antibiotics 25. Conversely, a report from China showed a low incidence of HP infection in the gastric conduit in patients who underwent esophagectomy, pyloroplasty, and reconstruction. The authors of that study concluded that this phenomenon was mostly due to the chronic reflux of bile after pyloroplasty 26.

Our findings were in concordance with previous reports on HP status and gastric tube cancer patients. In our Turkish population, HP infection was not associated with ESCC and had a similar pattern to that reported for Asian populations. HP infection may not contribute to the development of ESCC in patients who reside in rural areas of Asia, especially not in females. Furthermore, esophageal damage could be diminished by the prior presence of HP in areas with a high risk of ESCC. HP treatment has been associated with improved thrombocyte levels. An increase in platelet counts was observed in only 6.7% of treated patients. In the current study, we found no relationship between HP status and thrombocyte levels 27,28.

Finally, further studies are needed to address the impact of HP infection on ESCC and its natural history.

## AUTHOR CONTRIBUTIONS

Poyrazoglu OB was involved in developing the concept and design of the study and writing the manuscript. Dulger AC was involved in implementing the study and collecting data. Dulger AC and Gultepe BS were involved in collecting and processing the data. All the authors read and approved the final manuscript.

## Figures and Tables

**Table 1:** Descriptive statistics and comparison results according to Helicobacter pylori status.

	HP^1^	n	Mean	Std. Dev	Min.	Max.	*p*
Age (years)	+	65	57.02	11.514	32	92	0.239
	-	30	60.43	15.939	23	87	
	Total	95	58.09	13.084	23	92	
Hemoglobin (gr/dL)	+	63	12.50	2.025	8	17	0.371
	-	30	12.11	1.876	9	17	
	Total	93	12.38	1.976	8	17	
Hematocrit	+	63	37.20	5.616	24	49	0.427
	-	30	36.21	5.502	28	49	
	Total	93	36.88	5.569	24	49	
Leukocytes (/mm^3^)	+	63	7.598	3.8817	1.8	25.0	0.023
	-	30	5.803	2.4689	2.0	11.0	
	Total	93	7.019	3.5760	1.8	25.0	
Platelets (/mm^3^)	+	63	270.000	123.839	67.000	702.000	0.150
	-	30	616.687	324.542	79.000	178.000	
	Total	93	443.343	184.309	67.000	178.000	
ALT^2^ (U/L)	+	63	18.94	17.766	6	142	0.284
	-	30	15.27	8.026	6	39	
	Total	93	17.75	15.362	6	142	
AST^3^ (U/L)	+	62	33.02	48.837	2	341	0.089
	-	30	17.57	6.976	9	38	
	Total	92	27.98	40.833	2	341	
Albumin (g/dL)	+	58	3.71	0.734	2	5	0.882
	-	25	3.69	0.667	2	5	
	Total	83	3.71	0.711	2	5	
Globulin (g/dL)	+	47	3.07	0.533	2	4	0.045
	-	19	2.73	0.772	1	4	
	Total	66	2.97	0.625	1	4	
Calcium (mg/dL)	+	57	8.92	1.362	1	13	0.370
	-	27	9.17	0.639	8	11	
	Total	84	9.00	1.181	1	13	

**Std. Dev: Standard deviation**

1 Helicobacter pylori,

2 Alanine transaminase,

3 Aspartate transaminase
